# Development of Shortened Enrichment Methods for Detection of *Salmonella* Typhimurium Spiked in Milk

**DOI:** 10.1021/acsfoodscitech.2c00310

**Published:** 2023-04-21

**Authors:** Kevin Tsai, Matthew W Nonnenmann, Diane Rohlman, Kelly K. Baker

**Affiliations:** † Department of Occupational and Environmental Health, University of Iowa, Iowa City, Iowa 52246, United States; ‡ Department of Environmental, Agricultural and Occupational Health, University of Nebraska Medical Center, Omaha, Nebraska 69198, United States

**Keywords:** *Salmonella* Typhimurium, milk, food safety, enrichment, real-time PCR

## Abstract

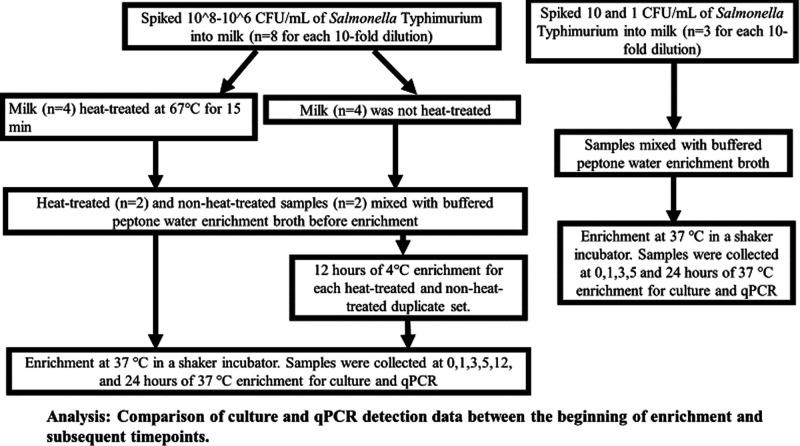

Rapid and accurate testing of pathogenic *Salmonella enterica* in dairy products could reduce the risk of exposure to the bacterial pathogens for consumers. This study aimed to reduce the assessment time needed for enteric bacteria recovery and quantification in food using the natural growth properties of *Salmonella enterica* Typhimurium (*S.* Typhimurium) in cow’s milk and efficiently using rapid PCR methods. Over 5 h of 37 °C enrichment, culture and PCR methods measured increases in the non-heat-treated *S.* Typhimurium concentration at similar rates, with an average increase of 2.7 log10 CFU/mL between the start of enrichment and 5 h. In contrast, no bacteria were recovered by culture after *S.* Typhimurium in milk received heat treatment, and the number of gene copies of heat-treated *Salmonella* detected by PCR did not increase with the enrichment time. Thus, comparing culture and PCR data over just 5 h of enrichment time can detect and differentiate between replicating bacteria and dead bacteria.

## 1 Introduction

Pathogenic *Salmonella* is one of the most common causes of foodborne illnesses. According to World Health Organization’s Foodborne Disease Burden Epidemiology Reference Group, pathogenic *Salmonella* caused 17 million foodborne illnesses, approximately 111 000 deaths, and 7.7 million disability-adjusted life years annually in 2010.^1^*Salmonella-*contaminated pasteurized milk has led to major foodborne outbreaks in the past,^2−4^ although a rise in demand for unpasteurized milk has also contributed to recent outbreaks. Cow’s milk, an essential nutrient source for growth and development globally, can be contaminated by pathogenic *Salmonella* from food-producing animals during the unsanitary production processes.^5,6^ Pasteurization is commonly used to eliminate the presence of *Salmonella* in raw milk as the method effectively kills most pathogenic *Salmonella* strains within seconds.^5^ However, *Salmonella* can also enter milk products by contact with *Salmonella*-contaminated containers and surfaces after pasteurization.^3,7^ These containers and surfaces become contaminated by water and soil containing human or animal feces with *Salmonella*.^7,8^ Laboratory monitoring of food product safety and the rapid response to threats are crucial for controlling foodborne pathogens and diseases. To reduce the occurrence of future *Salmonella* foodborne outbreaks, methods that rapidly detect viable *Salmonella* in cow milk at each risk point from farm to table should be a priority for public health.

Culture-based methods are considered to be the gold standard to detect enteric bacteria in food. However, culture-based methods are time-consuming and have low sensitivity, especially for environmentally stressed bacteria.^9,10^ Bacteria in food samples that experience physical and chemical stresses may be viable but not culturable (VBNC) or may replicate slowly in the first hours of culture.^11−13^ However, bacteria in the VBNC state can regain virulence and reproduce under favorable growth conditions in the mammalian gut.^14^ The failure to quickly detect VBNC bacteria in food safety assessments could cause an underestimation of food safety risk and allow unsafe foods into the market, where a no-tolerance policy for enteric pathogens is set as a milk safety standard in many countries.^15^

Food regulatory laboratories widely use enrichment methods to enhance the sensitivity of enteric bacteria detection assays before a selective culture. The method improves the recovery and detection of VBNC in foods that pose a risk to human health.^16,17^ The current standard Food and Drug Administration’s (FDA) protocol recommends a 24 to 72 h incubation at 37 °C in enrichment broth to improve bacterial detection sensitivity in food.^18,19^ However, the additional time needed for enrichment delays the investigation time needed for determining food safety and reduces the effectiveness of corresponding corrective actions. Unless serial dilutions of food are tested, culture results obtained from extended enrichment broths can only be used as presence/absence outcomes, preventing contamination levels from being correctly quantified. Quantitative data are crucial for predicting the risk of illness and mortality in a population exposed to contaminated foods. A rapid, sensitive, and quantitative food testing protocol could greatly improve the prevention and control of foodborne *Salmonella* and potentially other foodborne pathogens.

Colony counts of bacteria after cold enrichment could be used to estimate food contamination levels for some bacteria as cold temperatures slow down bacterial replication. Enrichment at 4 °C for 7 to 28 days coupled with enrichment at 37 °C for 18–24 h improved the overall recovery of *Yersinia enterocolitica* and *Listeria monocytogenes* in clinical samples and raw cow’s milk on culture.^20,21^ Brightwell and Clemens used cold enrichment at 7–10 °C for 3 weeks to increase the overall qPCR assay sensitivity of cold-tolerant *Clostridium estertheticum* in various types of environmental samples.^22^*Salmonella* responds differently to extended refrigeration in different food types, but previous research has shown that 3 days of refrigeration of food in enrichment broth ranging from 4 to 8 °C did not affect the recovery of *Salmonella* on culture.^23−28^ Shortening the enrichment time is another potential strategy for optimizing bacterial recovery and quantitative assessment. However, stressed bacteria may require more than 6 h of recovery time, depending on the type of food being tested and the type of microbial flora.^29−31^ Whether shortening the enrichment time enables quantitative assessment of pathogens in food is unclear. Kramer et al. found that qPCR with 8 h of warm enrichment significantly overestimated the number of *Salmonella* cells inoculated on pig carcasses compared to culture.^32^

Studies have utilized PCR’s higher sensitivity over the culture method to reduce the time needed for enrichment by eliminating the selective culturing step and coupling the enrichment along with real-time PCR (qPCR). Compared to nonenriched samples, qPCR sensitivity for detecting spiked *Salmonella enterica* (*S. enterica*), *Staphylococcus aureus, Listeria monocytogenes,* and *Escherichia coli* (*E. coli*) in raw milk and packaged meat improved after 3–4 h of warm enrichment in buffer peptone water or brain heart infusion broth at 37 °C.^33−35^ However, one of the criticisms of qPCR is that it may detect extracellular genes or genes from dead microbes and result in false positive interpretations. Currently, there is no consensus on how to eliminate false-positive results due to the detection of dead microbes in food, but developing a rapid qPCR method for the differentiation of viable and nonviable food contamination would be valuable. Additional research evaluating cold-enrichment or shortened enrichment processes, coupled with selective culture or qPCR, could address methodological gaps in the rapid quantification of *Salmonella* in milk.

The study aimed to develop a method that reduces the time needed to recover *Salmonella enterica* Typhimurium (*S.* Typhimurium) in milk, while allowing quantification by culture or qPCR. We hypothesized that allowing *Salmonella* to recover in enriched broth at 4 °C enrichment for 12 h followed by a 1 h 37 °C warm enrichment procedure would not negatively impact the time needed for qualitative and quantitative detection of *S.* Typhimurium in milk. We also hypothesized that repetitive cell culture and qPCR testing of 37 °C food enrichments could be applied to detect increases in *S.* Typhimurium concentrations over time. In the case of sequential qPCR testing, this would provide a faster and more sensitive means compared to the culture method in distinguishing between viable and nonviable *S.* Typhimurium. We tested these hypotheses using parallel selective culture and PCR methods to compare 24 h growth curves for heat-treated and non-heat-treated *S.* Typhimurium in milk during different enrichment conditions.

## 2 Materials and Methods

### 2.1 Assessment of Enrichment Performance Using Culture and qPCR

**Figure 1:**
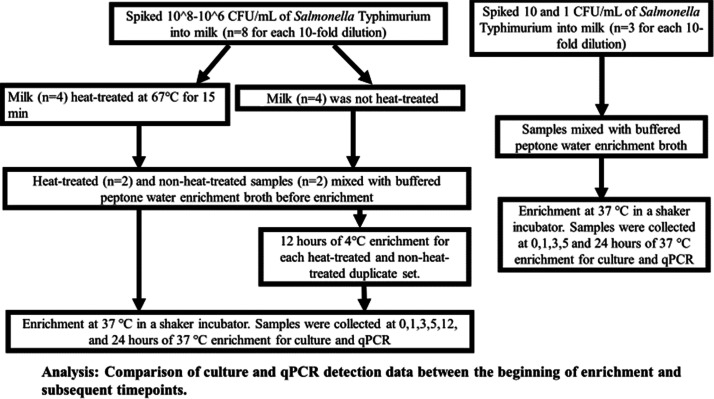
Overview of spiking, heat treatment, and enrichment procedures.

A 600 μL aliquot of live, overnight cultured *S.* Typhimurium DT104 (Catalog# BAA-190, ATCC, Manassas, VA), with concentration estimated and determined by OD600 spectrophotometer and plating, from three 10-fold serial dilutions (10^8^ to 10^6^ colony-forming unit (CFU)/mL) was spiked into 3 mL of pasteurized, previously frozen whole milk in a 10 mL conical centrifuge tube. The concentration of the spiking culture was determined by plating 10-fold dilutions of the culture on nonselective growth agar, identifying a dilution with countable colonies (20–200 CFU), averaging the colony count between duplicates, and then back calculating to the original CFU/ml. This approach resulted in a working concentration of 1.7 × 10^7^ to 1.7 × 10^5^ CFU/mL of milk. The spiking process was performed in four duplicate sets. Two duplicate spiked milk sets were heat-treated at 67 °C for 15 min in a hot water bath, while the other two were not heat-treated. The heat-shocking condition was used to emulate the lower-end pasteurization conditions (62 °C for 30 min) that could effectively damage or destroy *Salmonella* in dairy products.^36^ Afterward, the spiked milk sets were mixed at a 1:1 ratio with 3.6 mL of 1× buffered peptone water (BPW), resulting in a working concentration of 8.5 × 10^6^ to 8.5 × 10^4^ CFU/mL of *S.* Typhimurium in milk. Two heat-treated and two non-heat-treated spiked milk samples were enriched for 12 h at 4 °C plus 1 h at 37 °C, while the remaining sets were just incubated for 1 h at 37 °C, as described in Figure 1.

Bacterial culture and qPCR methods were used to measure the *S.* Typhimurium concentration of each enrichment scenario. The enriched *S.* Typhimurium with a working concentration at 1.7 × 10^6^ CFU/mL was 10-fold serially diluted with BPW to 10^2^ to 10^0^ CFU/mL for each spiked milk sample to isolate a dilution with quantifiable colonies. Then 200 μL of each serial dilution was spread plated on *Salmonella* selective MUG agar in duplicate (Catalog# 44782, Sigma-Aldrich, St. Louis, MO) and incubated for 24 h at 37 °C. For qPCR, a total of 200 μL of each spiked milk sample was collected into a Zymo DNA/RNA Shield lysis and collection tube (Catalog# R1103, Zymo Research, Irvine, CA), stored at −20 °C and bead beaten for 30 min in Vortex-Genie 2 mixer (Scientific Industries, Bohemia, NY) before extraction. With the manufacturer’s protocols, *S.* Typhimurium in enriched milk was extracted via the ZymoBIOMICS DNA/RNA Miniprep Kit (Catalog# R2202, Zymo Research, Irvine, CA). The qPCR for detecting *S.* Typhimurium extracts was performed with the TaqMan Fast Advanced Master Mix (Catalog# 4444556, Life Technology, Carlsbad, CA) in a QuantStudio7 qPCR system (Model# 4485701, Life Technology, Carlsbad, CA). A total of 2 μL of the sample was mixed with 10 μL of master mix and 1 μL of TaqMan primer/probe (Gene: *ttr*, forward primer: CTCACCAGGAGATTACAACATGG, reverse primer: AGCTCAGACCAAAAGTGACCATC, probe: FAM-CACCGACGGCGAGACCGACTT-NFQMGB)^37^ and 7 μL of nuclease-free water in a standard 96-well plate. The qPCR cycling conditions were as follows: hot-start stage at 95 °C for 2 min, and 40 cycles of 95 °C for 1 s and 60 °C for 20 s. Every sample was tested in duplicate by qPCR.

### 2.2 Assessment of *S.* Typhimurium Growth after 24 h Using qPCR

A total of 600 μL of live *S.* Typhimurium in the concentration of 10^7^ CFU/mL was spiked into 3 mL of pasteurized, previously frozen whole milk in a 10 mL conical centrifuge tube in duplicate. The spiked milk either underwent 15 min of heat treatment at 67 °C or without heat treatment. Afterward, the heat-treated and the non-heat-treated milk were mixed with 3 mL of BPW, resulting in a final concentration of 8.85 × 10^5^ CFU/mL of *S.* Typhimurium, then underwent 0, 24, 48, and 120 h of enrichment at 37 °C in a shaker incubator. The spiked milk at each target time was collected and extracted using the method described in the prior paragraph with a Zymo DNA/RNA Shield collection tube and Zymo Nucleic Acid Extraction Kit. The extracted nucleic acid templates were tested in duplicate with qPCR with *ttr* as the target gene by the qPCR method described previously.

### 2.3 Assessment of *S.* Typhimurium Growth under Low Concentration

A total of 600 μL of S. Typhimurium in concentrations of 10 and 1 CFU/mL was spiked into 3 mL of whole milk in triplicate. Then the spiked milk was mixed with 3 mL of BPW, resulting in a final concentration of 0.885 and 0.0885 CFU/mL of *S.* Typhimurium. The spiked milk sample with BPW underwent 0, 1, 3, 5, and 24 h of enrichment at 37 °C in a shaker incubator. The spiked milk with 10 CFU/mL *S.* Typhimurium at each target time was collected and extracted with Zymo DNA/RNA Shield collection tube and Zymo Nucleic Acid Extraction Kit and was tested in triplicate with qPCR with *ttr* as the target gene by the qPCR method described previously. A total of 200 μL of spiked milk at each enrichment time with starting *S.* Typhimurium concentrations of 0.885 and 0.0885 CFU/mL was also spread plated on *Salmonella* selective MUG agar and incubated overnight as well.

### 2.4 Statistical Analysis

For culture, bacteria concentrations were quantified by back-calculation of dilutions with countable CFU and then converted into log10 concentrations of *S.* Typhimurium in CFU/mL. To obtain denominators for comparing the culture and qPCR concentrations, the log10 CFU/mL concentration of *S.* Typhimurium detected by qPCR was calculated for each enrichment scenario from a qPCR standard curve constructed from *S.* Typhimurium with known concentrations (Figure S1). Each enrichment scenario’s arithmetic mean and standard deviation were calculated for all sample collection points for culture and qPCR, then tabulated or plotted as tables and figures.

The culture and qPCR concentrations of *S.* Typhimurium at 0 h of enrichment at 37 °C were compared by Student’s *t* test to determine whether there was a difference in recovery rate between the two detection methods. Student’s *t* test was also performed to determine whether there was a difference in *S.* Typhimurium concentrations between the method with cold enrichment and the method without cold enrichment and when the difference between the heat-treated and non-heat-treated *S.* Typhimurium population became significant. The data analysis was completed in SAS 9.4 and Microsoft Excel 2019.

## 3 Results and Discussion

### 3.1 Influence of Additional Cold Enrichment on the Growth of *Salmonella* Typhimurium in Milk

**Figure 2:**
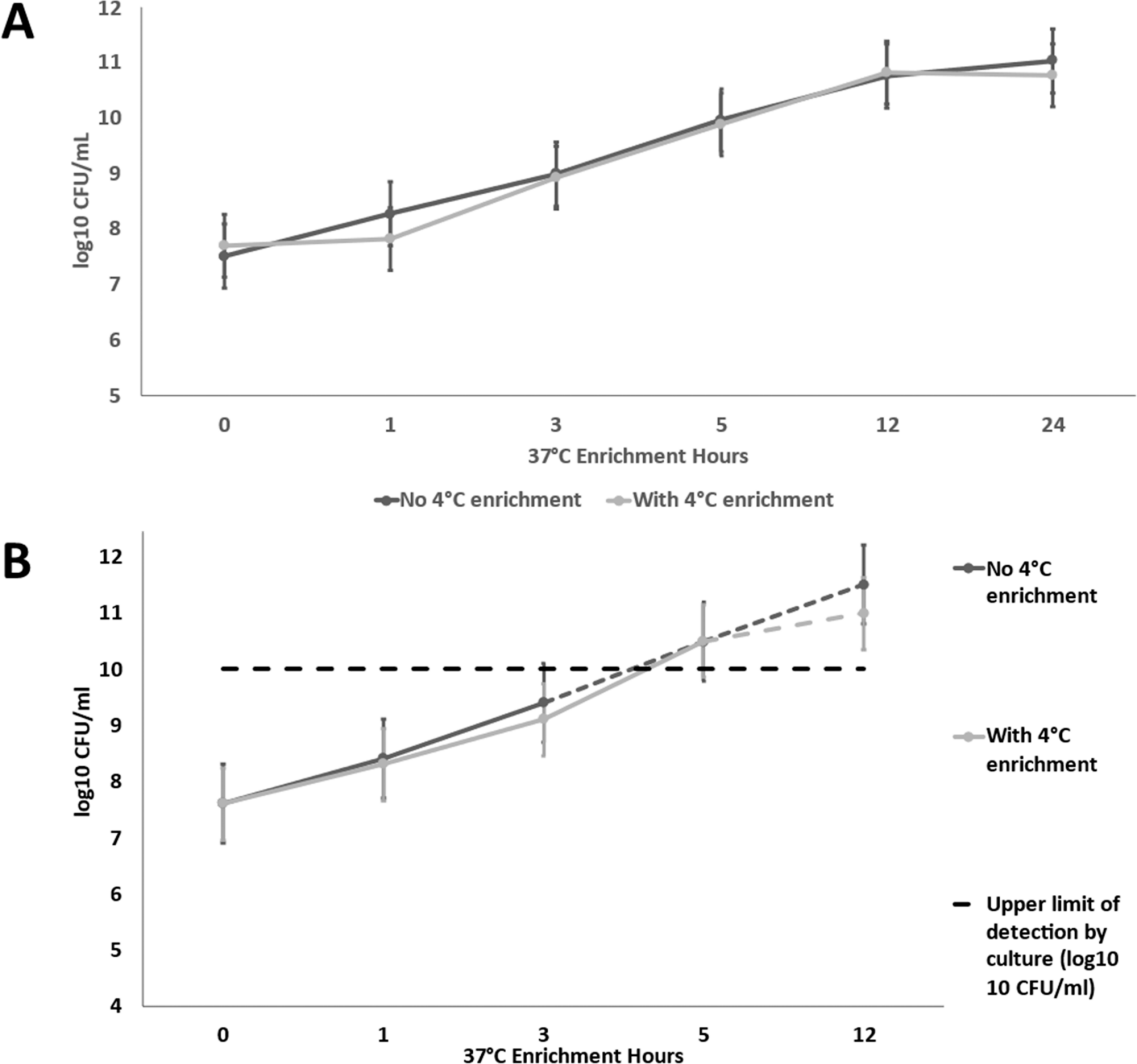
Growth of non-heat-treated *S.* Typhimurium with and without 4 °C enrichment procedure measured by (A) real-time PCR (each data point is the arithmetic mean and standard deviation of measurements (*n* = 6) and (B) selective culture (each data point is the arithmetic mean and standard deviation of measurements (*n* = 4); no recoverable *S.* Typhimurium was detected on culture after heat treatment).

The qPCR extraction efficiency was high, detecting more than 90% of *S.* Typhimurium spiked into the milk before the milk underwent 37 °C enrichment (Table 1). The qPCR assay was sensitive to 10^0^ CFU/μL, and the culture assay was sensitive up to 10^0^ CFU/mL (Figure S1). For the milk containing non-heat-treated *S.* Typhimurium, the qPCR estimated concentration of *S.* Typhimurium was nearly identical after 12 h of cold enrichment (time zero), indicating no gain or loss of bacterial cells. The concentration then increased by approximately three log10 CFU/mL between zero to 12 h of enrichment at 37 °C, both with and without 4 °C enrichment, indicating no influence of additional cold enrichment on replication rates (Figure 2A and Table S1). Like the qPCR results, the culture results indicated no difference in concentration after 12 h of cold enrichment, followed by an increase in *S.* Typhimurium concentration of at least three log10 CFU/mL after 12 h of enrichment at 37 °C, both with and without 4 °C enrichment, indicating no influence of additional cold enrichment on replication rates (Figure 2B and Table S2). The concentrations detected by culture and qPCR correlated well across time points.

**Table 1:** Percent of *S.* Typhimurium in 10^8^ to 10^6^ CFU/mL Recovered by qPCR from Spiked Milk at Zero h of Enrichment

**Concentration at the point of nucleic acid extraction**	**Recovery %**
6.8 × 10^6^ CFU/mL(*n* = 8)	96.4
6.8 × 10^5^ CFU/mL(*n* = 8)	95.8
6.8 × 10^4^ CFU/mL(*n* = 8)	97.9

### 3.2 Influence of Heat-Treatment Enrichment on the Growth of *Salmonella* Typhimurium in Milk

**Figure 3:**
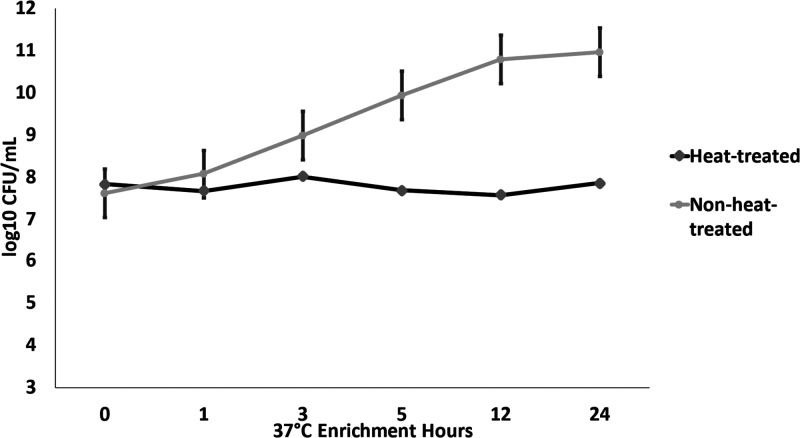
Growth of heat-treated and nontreated *S.* Typhimurium measured by qPCR. Each data point is the arithmetic mean and standard deviation of measurements (*n* = 12).

Since the number of *S.* Typhimurium cells detected by qPCR was not influenced by cold enrichment, the concentration results of cold-enriched and non-cold-enriched spiked milk were grouped to compare heat-treated and nonshocked spiked milk and identify the earliest time points where the change in bacterial concentrations revealed bacterial replication. *S.* Typhimurium was not isolated from any heat-treated *S.* Typhimurium spiked milk by culture assay, even after 12 h of 4 °C cold enrichment and 24 h of enrichment at 37 °C. While qPCR did indicate heat-treated milk was positive for *S.* Typhimurium, the detected concentrations did not vary more than one log10 CFU/mL between 0 to 24 h of enrichment at 37 °C (Figure 3 and Table S3). However, the difference in *S.* Typhimurium cells detected by qPCR in heat-treated versus non-heat-treated milk was statistically greater (*p* < 0.01) after 3 h of enrichment at 37 °C. This gene copy difference from enrichment onset expanded over time.

To test whether heat-treated bacteria could begin replicating and be recovered after 24 h of enrichment, such as in the case of VBNR state, we extended the recovery time by enriching milk at 37 °C beyond 24 h and repeating qPCR testing. Identical to culture results, which failed to recover any culturable *S.* Typhimurium, the milk with heat-treated *S.* Typhimurium failed to change in concentration detected by qPCR even after an excessive 4 days of enrichment at 37 °C (Table 2, *p*-value = 0.88). The non-heat-treated *S.* Typhimurium concentrations detected by qPCR remained unchanged between 24 to 120 h of 37 °C incubation (*p*-value = 0.46). The amount of *S.* Typhimurium concentrations detected by culture was above the assay’s limit of detection between 24 to 120 h of 37 °C incubation.

**Table 2:** Growth of Heat-Treated and Non-heat-treated *S.* Typhimurium in Milk after 120 h Measured by qPCR^a^

**Non-heat-treated**		**Heat-treated**	
**37 °C enrichment hours**	**log10 (CFU/mL)**	**37 °C enrichment hours**	**log10 (CFU/mL)**
0	7.9 ± 0.3	0	8.0 ± 0.1
24	10.9 ± 0.0	24	8.1 ± 0.1
48	10.7 ± 0.1	48	7.8 ± 0.1
120	10.7 ± 0.2	120	8.1 ± 0.5

a Arithmetic mean and standard deviation of measurements (*n* = 2).

### 3.3 Detection of Low-Concentration *S.* Typhimurium Growth by qPCR and Selective Culture

For the spiking concentration of 10 CFU/mL, *S.* Typhimurium was detectable by qPCR after 24 h of 37 °C enrichment and above the initial spiking concentration (24 h concentration: log10(CFU/mL) = 3.9 ± 0.4, *n* = 3) (Table 3). However, *S.* Typhimurium was detectable by culture after 1 h (10 CFU/mL spiking concentration) and 5 h (1 CFU/mL spiking concentration) of 37 °C enrichment, even though the number of *S.* Typhimurium became too numerous to count at both spiking concentrations.

**Table 3:** Growth of Low Concentration of *S.* Typhimurium in Milk after 120 h Measured by Selective Culture^a^

**10 CFU/mL**		**1 CFU/mL**	
**37 °C enrichment hours**	**log10 (CFU/mL)**	**37 °C enrichment hours**	**log10 (CFU/mL)**
0	No detection	0	No detection
1	0.69 ± 0.0	1	No detection
3	2.70 ± 0.1	3	No detection
5	≥3	5	2.56 ± 0.1
24	≥4	24	≥4

a Arithmetic mean and standard deviation of measurements (*n* = 3).

### 3.4 Discussion

As foodborne illnesses continue to affect communities globally, an alternative pathogen detection method that could overcome the time-consuming nature of the conventional culture and enrichment protocols would be more effective in preventing the spread of foodborne pathogens and foodborne illnesses.^10^ Using culture and qPCR approaches and leveraging the natural properties of enteric bacteria to replicate at 37 °C, we showed that reducing the 37 °C enrichment recovery time paired with qPCR testing at two sequential time points could expedite the detection of viable *S.* Typhimurium in milk in both low and high concentration contaminated milk. The improvement in overall qPCR sensitivity after a short warm enrichment time is similar to what Ding et al. demonstrated with raw milk and Garrido-Maestu et al. with raw meat.^33,34^ In this study, the *S. Typhimurium* concentrations do not noticeably change at 4 °C but proliferate in BPW/milk at 37 °C within just 3 to 5 h of enrichment, being sufficient to enable qPCR detection of bacterial growth. Compared with the traditional culture method, which requires several days to assess whether a sample contains viable pathogens, the two-time point qPCR method could reduce the time needed for viability assessment to within 24 h, while improving *Salmonella* detection sensitivity. In addition, the study also showed that the two-time point qPCR method could be used to confirm whether there is viable *S.* Typhimurium in milk.

Monitoring growth curves of non-heat-treated *S.* Typhimurium allowed us to estimate the recovery rate of *S.* Typhimurium in milk under various enrichment conditions. We expected that a 12 h enrichment at 4 °C, rather than an overnight enrichment at 37 °C, would optimize the recovery of injured *S.* Typhimurium in milk without eliminating the possibility of enumeration. Reducing enrichment time in the analysis process would also decrease the turnaround time for microbial testing results, thus reducing the duration of completion and intervention for food-related *Salmonella* microbial risk assessments. Using both culture and qPCR detection approaches, we did not find the milk enriched at 4 °C for 12 h contained a higher quantity of *S.* Typhimurium than the milk without 4 °C enrichment. However, this was likely due to a high recovery rate of viable *S.* Typhimurium. The 12 h enrichment at 4 °C did not seem to have an extended negative effect on the *S.* Typhimurium growth in this study. We did not observe a log10 concentration difference of more than one between the two enrichment procedures under the qPCR assay across various 37 °C enrichment time points.

The fact that the growth of *S.* Typhimurium at 4 °C did not significantly differ from the immediately enriched milk after 24 h of enrichment indicated that preserving samples via refrigeration for short periods also does not reduce the quality of quantitative analysis of *S. Typhimurium* concentrations. This observation agrees with past studies that 4 °C refrigeration has little effect on the survival of *S. enterica*, and the *S. enterica* cells may still proliferate, but at a slower rate.^38,39^ Although there was no detection enhancement from 12 h cold enrichment, the lack of growth curve difference between the refrigerated and the nonrefrigerated milk samples in the study demonstrated that refrigeration would have minimal impact on quantitative analysis results in cases where the food samples cannot be analyzed immediately. In fact, measuring the bacteria concentration change from the onset of 37 °C enrichment to either three to 5 h of 37 °C enrichment by qPCR rapidly identified viable *S.* Typhimurium where contamination was at least 10^3^ cells per mL in concentration. We found that the two-time point sampling process for qPCR was critical for a molecular viability assessment. Our longitudinal culture and qPCR attempts to detect viable *S.* Typhimurium indicated that pasteurization heating conditions, which we emulated, were sufficient to eliminate the spiked *S.* Typhimurium present in milk. We cultured no *S.* Typhimurium on the *Salmonella* selective MUG agar for any heat-treated *S.* Typhimurium spiked milk, even after 37 °C enrichment. While the qPCR assay consistently detected *S.* Typhimurium genes in heat-treated milk, no concentration change was observed over 24 h of warm enrichment, regardless of whether the milk had been cold enriched at 4 °C for 12 h. The lack of viable *S.* Typhimurium being observed on culture and qPCR after heat treatment agreed with the past studies that with sufficient time, pasteurization temperatures eliminate *S.* Typhimurium from dairy products.^40^ In comparison, the milk containing non-heat-treated *S.* Typhimurium experienced a minimum of 1000-fold concentration increase detectable by culture and qPCR after 24 h of 37 °C enrichment at low and high concentrations. Although viable *S.* Typhimurium was not observed on culture and qPCR in this study for the heat-treated milk, heat-resistant *S.* Typhimurium serotypes can persist in pasteurized food products after treatment and be recoverable by culture after overnight enrichment.^41,42^ Additional 37 °C enrichment time that is more than 10 h and addition PCR tests may be required to detect low concentration bacteria or recover the heat-injured but viable *Salmonella* in food.^43^ However, both the protocol we propose here and other qPCR-based assays still shorten the enrichment time needed to recover heat-injured *S.* Typhimurium presented in food compared with culture assays.^44,45^

Our observations of non-heat-shocked *S.* Typhimurium suggest that challenges persist when attempting to quantify *S.* Typhimurium after 24 h of warm enrichment. The *S.* Typhimurium concentrations were above the limit of quantification for both culture and qPCR methods after 24 h of 37 °C enrichment at high concentrations. Our results and previous research indicate that quantification of pathogens using the current U.S. Food and Drug Administration’s 24–72 h enrichment protocols could cause the overgrowth of pathogens, thus limiting the microbial risk assessment of enteric pathogens via multiday enrichment to qualitative interpretation.

Our observations support using the two-time point method for a quantitative microbial risk assessment. All of the non-heat-treated milk tested by culture in the study were above the limit of quantification (i.e., too numerous to count) overnight, while all non-heat-treated milk tested were quantifiable by qPCR. However, at the 1 h 37 °C enrichment mark, we found a wide concentration difference in the non-heat-treated *S.* Typhimurium by qPCR at high concentrations. We believe that the variance observed was likely due to inefficient amplification and subsampling error associated with lower target concentration when using qPCR.^46^ On the other hand, we found that the two-point culture method can be potentially used to supplement qPCR at lower concentrations when qPCR failed to detect microorganisms, as we were able to recover and observe significant changes in cell population via culture within the assay’s limit of detection using the two-time point method at low concentration.

Our plan for future studies is to increase the number of samples collected and analyze samples at lower concentrations to reduce the likelihood of inefficient amplification and subsampling error. Since we were not able to generate VBNC or nonviable *Salmonella,* we were not able to assess nucleic acid isolation, detection, and quantification for their growth curves via qPCR. Future investigations with different types of milk products using a similar enrichment and qPCR analysis approach with various types of heat treatment conditions could provide more understanding of the feasibility of quantifying *Salmonella* and other common enteric pathogens in milk using qPCR.

In conclusion, shortening the 37 °C enrichment time to 5 h, coupled with at least two sequential qPCR tests of a sample at enrichment onset (0 h) and 3 to 5 h, is a suitable approach for rapidly detecting pasteurized milk contamination by viable *S.* Typhimurium. Reducing the time needed to screen food samples could enable the prevention and control of foodborne diseases to be conducted in a timely matter. In addition, overnight refrigeration of food samples is methodologically acceptable in cases where they cannot be analyzed immediately. Quantifying the number of viable *Salmonella* in food samples can be achieved by the standard method of estimating the concentration measured before enrichment from a standard curve but then validating that concentration estimate by confirming an expected growth trend between enrichment times. We demonstrated this methodological approach for quantifying the number of viable *Salmonella* in cow milk due to the importance of this bacteria and food source in global health. However, future studies could explore the reproducibility of quantifying other enteric bacteria by qPCR and the validity of this approach compared to standard food safety protocols.

## Supporting Information Available

*S. enterica* DNA standard curve assessment methodology and results; growth of non-heat-treated *S*. Typhimurium with and without 4 °C enrichment measured by qPCR and culture; growth of heat-treated and non-heat-treated *S.* Typhimurium from 0 to 24 h of 37 °C enrichment measured by qPCR (PDF)

## References

[ref1] WHO WHO Estimates of the Global Burden of Foodborne Diseases; World Health Organization: Geneva, Switzerland, 2015.

[ref2] Ryan C. A.; Nickels M. K.; Hargrett-Bean N. T.; Potter M. E.; Endo T.; Mayer L.; Langkop C. W.; Gibson C.; McDonald R. C.; Kenney R. T. Massive outbreak of antimicrobial-resistant salmonellosis traced to pasteurized milk. JAMA 1987, 258 (22), 3269–3274. 10.1001/jama.1987.03400220069039. 3316720

[ref3] Olsen S. J.; Ying M.; Davis M. F.; Deasy M.; Holland B.; Iampietro L.; Baysinger C. M.; Sassano F.; Polk L. D.; Gormley B. Multidrug-resistant *Salmonella* Typhimurium infection from milk contaminated after pasteurization. Emerging Infectious Diseases 2004, 10 (5), 932 10.3201/eid1005.030484. 15200835 PMC3323239

[ref4] Control C. f. D. Salmonellosis From Inadequately Pasteurized milk--Kentucky. MMWR 1984, 33 (36), 505–506. 6433164

[ref5] Marth E. Salmonellae and salmonellosis associated with milk and milk products. A review. Journal of Dairy Science 1969, 52 (3), 283–315. 10.3168/jds.S0022-0302(69)86552-5. 4885967

[ref6] Karshima N.; Pam V.; Bata S.; Dung P.; Paman N. Isolation of *Salmonella* species from milk and locally processed milk products traded for human consumption and associated risk factors in Kanam, Plateau State, Nigeria. Journal of Animal Production Advances 2013, 3 (3), 69–74. 10.5455/japa.20130330124355.

[ref7] Carrasco E.; Morales-Rueda A.; García-Gimeno R. M. Cross-contamination and recontamination by *Salmonella* in foods: a review. Food Research International 2012, 45 (2), 545–556. 10.1016/j.foodres.2011.11.004.

[ref8] Forshell L. P.; Wierup M. *Salmonella* contamination: a significant challenge to the global marketing of animal food products. Rev. sci. tech. Off. int. Epiz 2006, 25 (2), 541–554. 10.20506/rst.25.2.1683. 17094696

[ref9] Mancini N.; Carletti S.; Ghidoli N.; Cichero P.; Burioni R.; Clementi M. The era of molecular and other non-culture-based methods in diagnosis of sepsis. Clin. Microbiol. Rev. 2010, 23 (1), 235–251. 10.1128/CMR.00043-09. 20065332 PMC2806664

[ref10] Law J. W.-F.; Ab Mutalib N.-S.; Chan K.-G.; Lee L.-H. Rapid methods for the detection of foodborne bacterial pathogens: principles, applications, advantages and limitations. Frontiers in Microbiology 2015, 5, 770 10.3389/fmicb.2014.00770. 25628612 PMC4290631

[ref11] Li L.; Mendis N.; Trigui H.; Oliver J. D.; Faucher S. P. The importance of the viable but non-culturable state in human bacterial pathogens. Frontiers in Microbiology 2014, 5, 258 10.3389/fmicb.2014.00258. 24917854 PMC4040921

[ref12] Schottroff F.; Fröhling A.; Zunabovic-Pichler M.; Krottenthaler A.; Schlüter O.; Jäger H. Sublethal injury and viable but non-culturable (VBNC) state in microorganisms during preservation of food and biological materials by non-thermal processes. Frontiers in Microbiology 2018, 9, 2773 10.3389/fmicb.2018.02773. 30515140 PMC6255932

[ref13] Garbeva P.; Hol W. G.; Termorshuizen A. J.; Kowalchuk G. A.; De Boer W. Fungistasis and general soil biostasis–a new synthesis. Soil Biology and Biochemistry 2011, 43 (3), 469–477. 10.1016/j.soilbio.2010.11.020.

[ref14] Du M.; Chen J.; Zhang X.; Li A.; Li Y.; Wang Y. Retention of virulence in a viable but nonculturable *Edwardsiella tarda* isolate. Appl. Environ. Microbiol. 2007, 73 (4), 1349–1354. 10.1128/AEM.02243-06. 17189433 PMC1828651

[ref15] Council N. R. Scientific Criteria to Ensure Safe Food; National Academies Press: Washington, D.C., 2003. 25057659

[ref16] Ray B. Impact of bacterial injury and repair in food microbiology: its past, present and future. Journal of Food Protection 1986, 49 (8), 651–655. 10.4315/0362-028X-49.8.651. 30959701

[ref17] Hoorfar J.; Baggesen D. L. Importance of pre-enrichment media for isolation of *Salmonella* spp. from swine and poultry. FEMS Microbiology Letters 1998, 169 (1), 125–130. 10.1111/j.1574-6968.1998.tb13308.x. 9851043

[ref18] Andrews W. H.; Jacobson A.; Hammack T., *Salmonella*. In Bacteriological Analytical Manual; U.S. Food and Drug Administration, 2011; Chapter 5.

[ref19] Feng P.; Weagant S. D.; Grant M. A.; Burkhardt W.; Shellfish M.; Water B., Enumeration of *Escherichia coli* and the Coliform Bacteria. In Bacteriological Analytical Manual; U.S. Food and Drug Administration, 2002; Vol. 13.

[ref20] Van Noyen R.; Vandepitte J.; Wauters G.; Selderslaghs R. *Yersinia enterocolitica*: its isolation by cold enrichment from patients and healthy subjects. Journal of Clinical Pathology 1981, 34 (9), 1052–1056. 10.1136/jcp.34.9.1052. 7024325 PMC494242

[ref21] Slade P. J.; Collins-Thompson D. Two-stage enrichment procedures for isolating *Listeria monocytogenes* from raw milk. Journal of Food Protection 1987, 50 (11), 904–908. 10.4315/0362-028X-50.11.904. 30978819

[ref22] Brightwell G.; Clemens R. Development and validation of a real-time PCR assay specific for *Clostridium estertheticum* and *C. estertheticum*-like psychrotolerant bacteria. Meat Science 2012, 92 (4), 697–703. 10.1016/j.meatsci.2012.06.025. 22782010

[ref23] He Y.; Chen R.; Qi Y.; Salazar J. K.; Zhang S.; Tortorello M. L.; Deng X.; Zhang W. Survival and transcriptomic response of Salmonella enterica on fresh-cut fruits. Int. J. Food Microbiol. 2021, 348, 109201 10.1016/j.ijfoodmicro.2021.109201. 33930836

[ref24] Lieberman V. M.; Zhao I. Y.; Schaffner D. W.; Danyluk M. D.; Harris L. J. Survival or growth of inoculated *Escherichia coli* O157: H7 and *Salmonella* on yellow onions (Allium cepa) under conditions simulating food service and consumer handling and storage. Journal of Food Protection 2015, 78 (1), 42–50. 10.4315/0362-028X.JFP-14-281. 25581176

[ref25] Pradhan A.; Li M.; Li Y.; Kelso L.; Costello T.; Johnson M. A modified Weibull model for growth and survival of *Listeria innocua* and *Salmonella* Typhimurium in chicken breasts during refrigerated and frozen storage. Poultry Science 2012, 91 (6), 1482–1488. 10.3382/ps.2011-01851. 22582310

[ref26] El-Gazzar F. E.; Marth E. H. Salmonellae, salmonellosis, and dairy foods: a review. Journal of Dairy Science 1992, 75 (9), 2327–2343. 10.3168/jds.S0022-0302(92)77993-4. 1452840

[ref27] Airoldi A.; Zottola E. Growth and survival of *Salmonella* typhimurium at low temperature in nutrient deficient media. J. Food Sci. 1988, 53 (5), 1511–1513. 10.1111/j.1365-2621.1988.tb09311.x.

[ref28] D’Aoust J.; Maishment C.; Burgener D.; Conley D.; Loit A.; Milling M.; Purvis U. Detection of *Salmonella* in refrigerated preenrichment and enrichment broth cultures. Journal of Food Protection 1980, 43 (5), 343–345. 10.4315/0362-028X-43.5.343. 30822867

[ref29] Zhao T.; Doyle M. P. Evaluation of universal preenrichment broth for growth of heat-injured pathogens. Journal of Food Protection 2001, 64 (11), 1751–1755. 10.4315/0362-028X-64.11.1751. 11726154

[ref30] Baylis C.; MacPhee S.; Betts R. Comparison of two commercial preparations of buffered peptone water for the recovery and growth of *Salmonella* bacteria from foods. J. Appl. Microbiol. 2000, 89 (3), 501–510. 10.1046/j.1365-2672.2000.01145.x. 11021583

[ref31] Chen H.; Fraser A. D.; Yamazaki H. Evaluation of the toxicity of *Salmonella* select media for shortening the enrichment period. Int. J. Food Microbiol. 1993, 18 (2), 151–159. 10.1016/0168-1605(93)90219-7. 8494681

[ref32] Krämer N.; Löfström C.; Vigre H.; Hoorfar J.; Bunge C.; Malorny B. A novel strategy to obtain quantitative data for modelling: combined enrichment and real-time PCR for enumeration of salmonellae from pig carcasses. Int. J. Food Microbiol. 2011, 145, S86–S95. 10.1016/j.ijfoodmicro.2010.08.026. 20855120

[ref33] Ding T.; Suo Y.; Zhang Z.; Liu D.; Ye X.; Chen S.; Zhao Y. A multiplex RT-PCR assay for *S. aureus*, *L. monocytogenes*, and *Salmonella* spp. detection in raw milk with pre-enrichment. Frontiers in Microbiology 2017, 8, 989 10.3389/fmicb.2017.00989. 28620364 PMC5449760

[ref34] Garrido-Maestu A.; Azinheiro S.; Roumani F.; Carvalho J.; Prado M. Application of Short Pre-enrichment, and Double Chemistry Real-Time PCR, Combining Fluorescent Probes and an Intercalating Dye, for Same-Day Detection and Confirmation of Salmonella spp. and *Escherichia coli* O157 in Ground Beef and Chicken Samples. Frontiers in Microbiology 2020, 11, 2540 10.3389/fmicb.2020.591041. PMC7581864 33162968

[ref35] Fachmann M. S. R.; Löfström C.; Hoorfar J.; Hansen F.; Christensen J.; Mansdal S.; Josefsen M. H. Detection of *Salmonella enterica* in meat in less than 5 h by a low-cost and noncomplex sample preparation method. Appl. Environ. Microbiol. 2017, 83 (5), e03151 10.1128/AEM.03151-16. 27986726 PMC5311390

[ref36] Juffs H. S.; Deeth H. Scientific Evaluation of Pasteurisation for Pathogen Reduction in Milk and Milk Products; Food Standards Australia New Zealand: Canberra, Australia, 2007.

[ref37] Liu J.; Gratz J.; Amour C.; Nshama R.; Walongo T.; Maro A.; Mduma E.; Platts-Mills J.; Boisen N.; Nataro J. Optimization of quantitative PCR methods for enteropathogen detection. PLoS One 2016, 11 (6), e0158199 10.1371/journal.pone.0158199. 27336160 PMC4918952

[ref38] Matches J. R.; Liston J. Low temperature growth of *Salmonella*. J. Food Sci. 1968, 33 (6), 641–645. 10.1111/j.1365-2621.1968.tb09092.x.

[ref39] D’Aoust J.-Y. Psychrotrophy and foodborne *Salmonella*. Int. J. Food Microbiol. 1991, 13 (3), 207–215. 10.1016/0168-1605(91)90004-9. 1892738

[ref40] Doyle M. E.; Mazzotta A. S. Review of studies on the thermal resistance of Salmonellae. Journal of Food Protection 2000, 63 (6), 779–795. 10.4315/0362-028X-63.6.779. 10852574

[ref41] D’Aoust J.-Y.; Emmons D.; McKellar R.; Timbers G.; Todd E.; Sewell A.; Warburton D. Thermal inactivation of *Salmonella* species in fluid milk. Journal of Food Protection 1987, 50 (6), 494–501. 10.4315/0362-028X-50.6.494. 30965449

[ref42] Shachar D.; Yaron S. Heat tolerance of *Salmonella enterica* serovars Agona, Enteritidis, and Typhimurium in peanut butter. Journal of Food Protection 2006, 69 (11), 2687–2691. 10.4315/0362-028X-69.11.2687. 17133812

[ref43] Stephens P.; Joynson J.; Davies K.; Holbrook R.; Lappin-Scott H.; Humphrey T. The use of an automated growth analyser to measure recovery times of single heat-injured *Salmonella* cells. J. Appl. Microbiol. 1997, 83 (4), 445–455. 10.1046/j.1365-2672.1997.00255.x. 9351226

[ref44] Mercanoglu B.; Griffiths M. W. Combination of immunomagnetic separation with real-time PCR for rapid detection of *Salmonella* in milk, ground beef, and alfalfa sprouts. Journal of Food Protection 2005, 68 (3), 557–561. 10.4315/0362-028X-68.3.557. 15771182

[ref45] Zheng Q.; Mikš-Krajnik M.; Yang Y.; Xu W.; Yuk H.-G. Real-time PCR method combined with immunomagnetic separation for detecting healthy and heat-injured *Salmonella* Typhimurium on raw duck wings. Int. J. Food Microbiol. 2014, 186, 6–13. 10.1016/j.ijfoodmicro.2014.06.005. 24974274

[ref46] Taylor S. C.; Nadeau K.; Abbasi M.; Lachance C.; Nguyen M.; Fenrich J. The ultimate qPCR experiment: producing publication quality, reproducible data the first time. Trends Biotechnol. 2019, 37 (7), 761–774. 10.1016/j.tibtech.2018.12.002. 30654913

