# Supplementary material for: Development of Shortened Enrichment Methods for Detection of Salmonella Typhimurium Spiked in Milk
Source: ACS Food Sci Technol. 2023 Apr 21;3(5):831–7. doi: 10.1021/acsfoodscitech.2c00310 (PMC10204049; doi:10.1021/acsfoodscitech.2c00310)
Supplement: Supplementary file 1 — fs2c00310_si_001.pdf [file fs2c00310_si_001.pdf]

Supplementary Material Corresponded to Article

S. enterica DNA standard curve assessment with TaqMan Fast Advanced Master Mix

The *S. enterica* nucleic acid ten-fold serial dilution extracts (Catalog# NR-514, BEI Resources, VA, USA) ranged from 5*10^4 to 10^0 CFU/µL were tested in a 96-well plate to create a qPCR standard curve. The qPCR run was completed in duplicates. For each well, 2 µL of the sample was mixed with 10 µL of TaqMan Fast Advanced Master Mix (Catalog# 4444556, Life Technology, CA, USA), 1 µL of *ttr* gene TaqMan primer (Forward primer: CTCACCAGGAGATTACAACATGG. reverse primer. AGCTCAGACCAAAAGTGACCATC, probe: FAM-CACCGACGGCGAGACCGACTT-NFQMGB), and 7 µL of nuclease-free water. The qPCR run was completed in a QuantStudio7 real-time PCR system (Model# 4485701, Life Technology, CA, USA). The qPCR cycling conditions were as follows: holding stage 95 °C for 2 minutes, and 40 cycles of 95 °C for 1 second and 60 °C for 20 seconds. The process was repeated in duplicate for each ten-fold serial dilution.

Supplemental Tables and Figures:

Table S1: The growth of non-heat-treated *S.* Typhimurium with and without 4°C enrichment measured by qPCR (in log10 CFU/mL)*.

	**37 °C enrichment hours**					
	**0**	**1**	**3**	**5**	**12**	**24**
**Without 4°C enrichment**	7.5 ± 0.2	8.3 ± 1.3	9.0 ± 0.1	10.0 ± 0.3	10.8 ± 0.9	11.0 ± 1.2
**With 4°C enrichment**	7.7 ± 0.3	7.8 ± 0.1	8.9 ± 0.2	9.9 ± 0.9	10.8 ± 0.9	10.8 ± 0.9
**P-value**	0.32	0.40	0.56	0.07	0.29	0.20

* Arithmetic mean and standard deviation of measurements (n=6) reported in log 10 CFU/mL estimated by comparison to *S.* Typhimurium standard curve.

Table S2: The growth of non-heat-treated *S.* Typhimurium with and without 4°C enrichment procedure measured by selective culture (in log10 CFU/mL)*.

	**37 °C enrichment hours**					
	**0**	**1**	**3**	**5**	**12**	**24**
**Without 4°C enrichment**	7.6 ± 0.1	8.4 ± 0.4	9.4 ± 0.3	≥ 10	≥ 10	≥ 10
**With 4°C enrichment**	7.6 ± 0.2	8.3 ± 0.4	9.1 ± 0.4	≥ 10	≥ 10	≥ 10
**p-value**	0.81	0.77	0.48			

*Arithmetic mean and standard deviation of all measurements (n=4) reported in CFU/mL.

Table S3: The growth of heat-treated and non-heat-treated *S.* Typhimurium from 0 to 24 hours of 37 °C enrichment measured by qPCR (in log10 CFU/mL)*.

	**37 °C enrichment hours**					
	**0**	**1**	**3**	**5**	**12**	**24**
**Heat-treated**	7.8 ± 0.1	7.7 ± 0.5	8.0 ± 0.3	7.7 ± 0.4	7.6 ± 0.3	7.9 ± 0.2
**Non-heat-treated**	7.6 ± 0.3	8.1 ± 0.9	9.0 ± 0.1	9.9 ± 0.3	10.8 ± 0.9	11.0 ± 1.0
**p-value**		0.12	< 0.01	< 0.01	< 0.01	< 0.01

*Arithmetic mean and standard deviation of measurements (n=12) reported in CFU/mL.

Figure S1: *S. enterica* DNA standard curve from 10^4 to 10^0 CFU/µL*

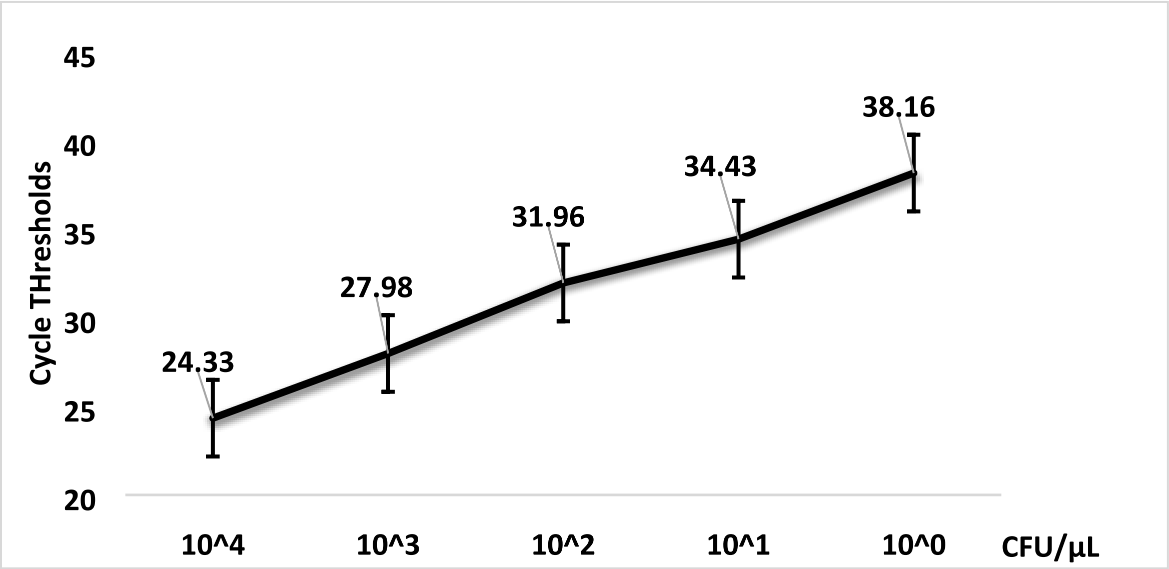

*Each point is the arithmetic mean of measurements (n=2)
